# Nitrogen source influences the interactions of comammox bacteria with aerobic nitrifiers

**DOI:** 10.1128/spectrum.03181-23

**Published:** 2024-03-21

**Authors:** Katherine Jeanne Vilardi, Juliet Johnston, Zihan Dai, Irmarie Cotto, Erin Tuttle, Ariana Patterson, Aron Stubbins, Kelsey J. Pieper, Ameet J. Pinto

**Affiliations:** 1 Department of Civil and Environmental Engineering, Northeastern University, Boston, Massachusetts, USA; 2 School of Civil and Environmental Engineering, Georgia Institute of Technology, Atlanta, Georgia, USA; 3 Department of Marine and Environmental Sciences, Northeastern University, Boston, Massachusetts, USA; 4 Department of Chemistry and Chemical Biology, Northeastern University, Boston, Massachusetts, USA; 5 School of Earth and Atmospheric Sciences, Georgia Institute of Technology, Atlanta, Georgia, USA; University of Mississippi, University, Mississippi, USA

**Keywords:** comammox, urea, nitrifiers, ammonia inhibition

## Abstract

While the co-existence of comammox *Nitrospira* with canonical nitrifiers is well documented in diverse ecosystems, there is still a dearth of knowledge about the mechanisms underpinning their interactions. Understanding these interaction mechanisms is important as they may play a critical role in governing nitrogen biotransformation in natural and engineered ecosystems. In this study, we tested the ability of two environmentally relevant factors (nitrogen source and availability) to shape interactions between strict ammonia and nitrite-oxidizing bacteria and comammox *Nitrospira* in continuous flow column reactors. The composition of inorganic nitrogen species in reactors fed either ammonia or urea was similar during the lowest input nitrogen concentration (1 mg-N/L), but higher concentrations (2 and 4 mg-N/L) promoted significant differences in nitrogen species composition and nitrifier abundances. The abundance and diversity of comammox *Nitrospira* were dependent on both nitrogen source and input concentrations as multiple comammox *Nitrospira* populations were preferentially enriched in the urea-fed system. In contrast, their abundance was reduced in response to higher nitrogen concentrations in the ammonia-fed system. The preferential enrichment of comammox *Nitrospira* in the urea-fed system could be associated with their ureolytic activity calibrated to their ammonia oxidation rates, thus minimizing ammonia accumulation, which may be partially inhibitory. However, an increased abundance of comammox *Nitrospira* was not associated with a reduced abundance of nitrite oxidizers in the urea-fed system while a negative correlation was found between them in the ammonia-fed system, the latter dynamic likely emerging from reduced availability of nitrite to strict nitrite oxidizers at low ammonia concentrations.

**IMPORTANCE:**

Nitrification is an essential biological process in drinking water and wastewater treatment systems for treating nitrogen pollution. The discovery of comammox *Nitrospira* and their detection alongside canonical nitrifiers in these engineered ecosystems have made it necessary to understand the environmental conditions that regulate their abundance and activity relative to other better-studied nitrifiers. This study aimed to evaluate two important factors that could potentially influence the behavior of nitrifying bacteria and, therefore, impact nitrification processes. Column reactors fed with either ammonia or urea were systematically monitored to capture changes in nitrogen biotransformation and the nitrifying community as a function of influent nitrogen concentration, nitrogen source, and reactor depth. Our findings show that with increased ammonia availability, comammox *Nitrospira* decreased in abundance while nitrite oxidizers abundance increased. Yet, in systems with increasing urea availability, comammox *Nitrospira* abundance and diversity increased without an associated reduction in the abundance of canonical nitrifiers.

## INTRODUCTION

Comammox bacteria, which belong to the genus *Nitrospira*, are routinely detected alongside strict ammonia-oxidizing bacteria (AOB) and nitrite-oxidizing bacteria (NOB) in both drinking water and wastewater systems (1–9), but insights into the factors influencing their abundance, activity, and interactions in these environments are still limited. Interactions between AOB and NOB have been extensively studied including the impact of operational processes and environmental conditions such as oxygen supply, ammonia concentration, and temperature (10–12). However, the presence of comammox *Nitrospira* within these communities requires a re-evaluation of these interactions and the collective response of nitrifying consortia to changes in environmental and/or process conditions. Our understanding of the role and ecological niche of comammox *Nitrospira* within complex nitrifying communities is further restricted by limited physiological insights due to the existence of only a few cultured representatives and/or enrichments, all belonging to clade A1 (13–15).

Ammonia availability is likely an important factor governing interactions between strict AOB and comammox *Nitrospira*. For instance, comammox *Nitrospira* cultures and enrichments have shown a significantly higher affinity for ammonia compared to strict AOB (14–16). Thus, comammox *Nitrospira* may outcompete strict AOB in ammonia-limited environments such as drinking water systems. Further, different comammox *Nitrospira* may exhibit varying preferences for ammonia concentration ranges, and these may not only be dictated by ammonia affinities but also include potential inhibition at higher concentrations. For example, ammonia oxidation by *Ca*. *Nitrospira kreftii* was partially inhibited at relatively low ammonia concentrations (25 µM) (15), which was not observed for *Ca*. *Nitrospira inopinata* (16). Comammox *Nitrospira* may also exhibit clade/sub-clade-dependent preferences for ammonia availability and/or environments. For example, clade A1 comammox *Nitrospira* associated with *Ca*. *Nitrospira nitrosa* are typically found at higher abundances than canonical nitrifiers in some wastewater systems (2, 7, 17, 18) and sometimes as the principal aerobic ammonia oxidizers in these systems (19). In the latter situation, ammonia oxidation dominated by comammox *Nitrospira* could also adversely impact *Nitrospira*-NOB by limiting nitrite availability through complete nitrification to nitrate at low ammonia concentrations; however, the relationship between the two *Nitrospira* groups is not well understood.

The nitrogen source could also have a significant effect on interactions between nitrifiers. For instance, ureolytic activity may enable access to ammonia derived from urea in engineered systems (e.g., wastewater treatment), as well as natural systems such as freshwater ecosystems (20). Genes for urea degradation accompanied by a diverse set of urea transporters are ubiquitously found in genomes of all comammox *Nitrospira* (21). Their ability to grow in urea is supported by the enrichment of multiple species of comammox *Nitrospira* in urine-fed membrane bioreactors (22) and the enrichment of comammox *Nitrospira* when supplied with urea (22, 23). Some *Nitrospira*-NOB are also capable of catalyzing ammonia production through urea degradation and, thus, potentially regulating nitrite availability via cross-feeding of ammonia to strict AOB (24); this could potentially influence competition between canonical nitrifiers and comammox *Nitrospira*.

The present work aimed to investigate the potential competitive interactions of comammox *Nitrospira* with canonical nitrifiers subject to different nitrogen sources and concentrations. We operated two continuous-flow laboratory-scale column reactors with granular activated carbon (GAC) containing all three nitrifying groups and supplied the reactors with either ammonia or urea at three different influent nitrogen concentrations. Our goal was to infer (i) nitrogen source (i.e., ammonia and urea), species (i.e., urea, ammonia, and nitrite), and concentration preferences of nitrifying groups and (ii) their potential interactions by quantitatively measuring their differential sorting within column reactors over time in the context of their genome-resolved metabolic capabilities.

## MATERIALS AND METHODS

### Reactor operation

Two laboratory-scale down-flow column reactors (diameter = 2.54 cm, height = 25.4 cm) were packed with GAC (packed height = 7.62 cm) and operated at room temperature (20°C–22°C) with an approximately 2.54 cm of water head above the GAC to ensure the media was fully saturated. The systems were each packed with 35 g of GAC from the City of Ann Arbor, Michigan Drinking Water Treatment Plant (DWTP). The two reactors were fed with synthetic groundwater media (25). Stock solution for the inorganic compounds in the media was prepared with 3.88 g/L MgCl_2_, 2.81 g/L CaCl_2_, 13.68 g/L NaCl, 6.90 g/L K_2_CO_3_, 17.75 g/L Na_2_SO_4_, and 0.88 g/L KH_2_PO_4_. The organic compound stock solution contained 3.75 g/L of glucose (C_6_H_12_O_8_), and a sodium bicarbonate solution was prepared with 30 g/L of NaHCO_3_. Influent media was then prepared in 10 L autoclaved carboys with 1 mL/L of the inorganic and organic compound stock solutions and 10 mL/L of the sodium bicarbonate stock solution. The two reactors were fed influent amended with stock solutions of ammonium chloride (NH_4_Cl) or urea (CH_4_N_2_O). Both reactors were fed influent at three different nitrogen concentrations over the experimental period. Column reactors were maintained in conditions 1 (1 mg-N/L) and 2 (2 mg-N/L) for 8 weeks and in condition 3 (4 mg-N/L) for 6 weeks. Influent media was pumped at 1.15 L/day with the peristaltic pump resulting in an empty bed contact time (EBCT) of approximately 48 minutes.

### Sample collection and processing

Influent and effluent were sampled twice weekly, while five samples spaced approximately 1.27 cm apart along the depth of the GAC column were collected weekly to capture depth-wise nitrogen species concentrations. The five sections are defined as sections 1.27 (L1), 2.54 (L2), 3.81 (L3), 5.08 (L4), and 6.35 (L5) cm (Fig. S1). All aqueous samples were filtered through 0.22-µm filters (Sartorius Minisart NML Syringe Filter—Fisher Scientific 14555269). GAC media samples were collected at week 0 followed by weeks 6, 7, and 8 for conditions 1 and 2 and week 6 for condition 3. GAC media samples (0.3 g) were collected from three locations along the reactor bed: one within the top 1.27 cm of the reactor, one mid-filter depth (3.81 cm), and another at the bottom approximately 7.62 cm from the top of the reactor location and were stored for DNA extraction in Lysing Matrix E Tubes (MP Biomedical—Fisher Scientific MP116914100). After each sampling event, the amount of GAC taken was replaced with virgin GAC, which was mixed with the remaining GAC by first fluidizing the filter media with 50 mL deionized water followed by backwashing with air for 5 minutes. A total of 42 GAC media samples were collected from seven sampling events. During each sampling event, we collected samples from three depths (top, L1; middle, L3; bottom, L5) for each of the two columns (Fig. S1). These GAC media samples were immediately stored at −80°C until further processing.

### Chemical analysis

Hach TNT Vials were used to determine concentrations of ammonia (TNT832), nitrite (TNT839), nitrate (TNT835), and total alkalinity (TNT870). All samples were analyzed on a Hach DR1900 photospectrometer (Hach—DR1900-01H). Influent and effluent pH was determined using a portable pH meter (Thermo Scientific Orion Star A221 Portable pH Meter—Fisher Scientific 13-645-522). A Shimadzu TOC-L (total organic carbon analyzer) with a TNM-L attachment (total nitrogen unit) (26) was used to measure total dissolved nitrogen in influent and effluent samples using certified DOC/TDN standards [deep seawater reference: low carbon seawater, LSW, deep seawater reference material] (Batch 21 Lot 11-21, 1). Urea concentrations in samples collected from the urea-fed reactors were determined by subtracting the total inorganic nitrogen measured (i.e., sum of ammonia, nitrite, and nitrate) in each sample from influent urea concentration.

### Nitrogen biotransformation rate calculations

Rates of nitrogen biotransformations were calculated from the concentration profiles of ammonia, NO_x_ (nitrite plus nitrate), nitrate, and total inorganic nitrogen (sum of ammonia, nitrite, and nitrate) measured along the column reactor depths. Rates were calculated for six sections of the columns: 0–1.27 (In-L1), 1.28–2.54 (L1-L2), 2.55–3.81 (L2-L3), 3.82–5.08 (L3-L4), 5.09–6.35 (L4-L5), and 6.36–7.62 (L5-Eff) cms. Volumetric rates (mg-N/L packed GAC/h) were obtained by multiplying the concentration differences between the profile layers by the influent flow rate and dividing by the volume of packed GAC between the profile layers (*V* = 6.4 mL packed GAC in each layer).

### DNA extraction and qPCR assays

DNA was extracted from all GAC samples (*n* = 43), which included the inoculum and samples collected at all time points and locations for each condition. Extractions were performed using Qiagen’s DNeasy PowerSoil Pro (Qiagen, Inc—Cat. No. 47014) with a few modifications. GAC in lysing matrix tubes with 800 µL of CD1 was vortexed briefly and placed in a 65°C water bath for 10 minutes. After heating, 500 µL of phenol:chloroform:isoamyl alcohol (25:24:1, vol/vol) (Invitrogen UltraPure—Fisher Scientific 15-593-031) was added to the lysing tube bead beating and processed with four 40-second rounds on the FastPrep-24 instrument (MP Biomedical—Cat. No. 116005500) with lysing tubes placed on ice for 2 minutes between rounds. Samples were then centrifuged for 1 minute, and 600 µL of the aqueous phase was used for DNA extractions on the Qiacube (Qiagen, Inc—Cat No. 9002160) protocol for PowerSoil Pro. A reagent blank was included in each round of extractions as a negative control. DNA concentrations were measured using a Qubit with the dsDNA Broad Range Assay (Invitrogen—Fisher Scientific Q32850). Extracted DNA was stored at −80°C until further processing.

Quantitative polymerase chain reaction (qPCR) assays were conducted using the Applied Biosystems 7500 Fast Real-Time PCR instrument. Primer sets listed in Table S1 were used to target the 16S rRNA gene of AOB (27), 16S rRNA gene of *Nitrospira* (28), *amoB* gene of clade A comammox *Nitrospira* (1), and 16S rRNA gene of total bacteria (29). The qPCR reactions were performed in 20 µL volumes, which contained 10 µL Luna Universal qPCR mastermix (New England Biolabs Inc., Fisher Scientific Cat. No. NC1276266), 5 µL of 10-fold diluted template DNA, primers at concentrations listed in Table S1, and DNA/RNAse-free water (Fisher Scientific, Cat. No. 10977015) to make the remaining volume. Each sample was subjected to qPCR in triplicate. The cycling conditions consisted of initial denaturing at 95°C for 1 minute, 40 cycles of denaturing at 95°C for 15 seconds, annealing times and temperatures listed in Table S1, and extension at 72°C for 1 minute. Three different sets of gBlock standards (Integrated DNA Technology gBlocks Gene Fragments 125–500 bp) targeting the 16S rRNA gene of total bacteria and *Nitrospira*, 16S rRNA gene of AOB, and *amoB* gene of clade A comammox *Nitrospira* were used to establish a seven-point standard curve for each respective assay (Table S2). The qPCR efficiencies for all assays are listed in Table S1.

### 16S rRNA gene amplicon sequencing and data analysis

DNA extracts (triplicate per sample) from all samples were submitted for sequencing of the V4 hypervariable region of the 16S rRNA gene at the Georgia Institute of Technology Sequencing Core. The MiSeq v2 kit was used to generate 250-bp pair-end reads using the 515F (30) and 806R (31) primers with an overhang of barcoded Illumina adapters. Removal of adapter and primer sequences from the resultant sequencing data was carried out using cutadapt v4.2. Amplicon sequencing data processing and quality filtering were performed using DADA2 v1.22.0 (32) in R v4.1.2. to infer amplicon sequence variants (ASVs) using the pipeline for paired-end Illumina MiSeq data. The SILVA nr v.138.1 database (33) was used for the taxonomic assignment of ASVs with a minimum bootstrap confidence threshold of 80. The ASV table was rarefied with the “rarefy_even_depth” function from the R package phyloseq v1.38.0 to the sample with the smallest library size. The relative abundance of ASVs in each sample was calculated by dividing ASV read counts in the sample by the total number of sample read counts.

### Metagenomic sequencing, assembly, and binning

DNA extracted from samples taken at week 6 from the top layer of the ammonia- and urea-fed reactors during condition 3 were submitted for sequencing on the Illumina NovaSeq platform with a SP flow cell at the Georgia Institute of Technology Sequencing Core. Similar workflows and tools utilized in references (1, 2) were applied here to assemble and characterize metagenome-assembled genomes (MAGs). Briefly, raw paired-end reads were quality-filtered using fastp (v0.22.0) (34) and further mapped to the Univec database to remove contaminant reads. Samtools (v1.15.1) (35) was used to sort the resulting bam files, and bedtools (v2.30.0) (36) was used to convert them to fastq files. Assemblies were generated for each sample separately using metaSpades (v3.13.0) (37) with kmer sizes 21, 33, 55, and 77. The two resulting fasta assemblies were indexed with bwa index (v0.7.17), and filtered pair-ended reads were mapped back to their respective assemblies with bwa mem (v0.7.17) (38). The subsequent sam files were converted to bam files using appropriate samtools (v1.15.1) flags to retain only mapped reads.

Metabat2 (39) was used to bin contigs longer than 2,000 bp followed by CheckM (v1.1.2) (40) to determine the completeness and contamination levels of MAGs, which were then classified using the Genome Taxonomy Database Tool Kit (v1.1.1) with database release 207 (41). Open reading frames of coding regions predicted using prodigal (42) were annotated against the KEGG database (43) with kofamscan (v1.2.0) (44). The up-to-date Bacterial Core Gene pipeline (v3.0) (45) was used to construct maximum likelihood trees using a set 92 extracted and aligned single-copy genes from assembled *Nitrospira*-like and *Nitrosomonas-*like MAGs and references. A set of dereplicated MAGs was generated from MAGs recovered from the ammonia- and urea-fed systems at an average nucleotide identity (ANI) threshold of 99% using dRep (v3.4.0) (46). Reads from the ammonia- and urea-fed samples were mapped to the set of dereplicated MAGs to calculate the breadth of coverage (i.e., percent of genome covered by reads) and relative abundance using coverM (https://github.com/wwood/CoverM).

16S rRNA gene sequences of a minimum length of 500 bp were reconstructed from the metagenomes of both samples using MATAM v1.6.1 (47) to establish the linkage between ASVs generated from 16S rRNA gene amplicon sequencing and MAGs associated with nitrifying bacteria. To achieve a more comprehensive reconstruction, recursive random sub-sampling of different depths, i.e., 1%, 5%, 10%, 25%, 50%, 75%, and 100%, was performed, followed by dereplication at 99.9% identity using USEARCH v11.0.667 (48, 49). Only the longest sequence from each cluster was retained for downstream analysis. Furthermore, ASVs were aligned against the MATAM recovered 16S rRNA gene sequences and extracted 16S rRNA genes from MAGs by Barrnap v0.9 using BLASTn v2.13.0 (50), and only ASV hits of 100% identity and 100% coverage were considered as linkage candidates between ASV and MAG unless the alignment was interrupted at the end of the reference sequence.

### Statistical analysis

All statistical analysis was performed in R v4.1.2 (51). A significant difference in effluent concentrations of ammonia, nitrite, and nitrate in the ammonia- and urea-fed systems was tested using the non-parametric Wilcox rank sum test. Ratios of effluent NO_x_ to influent nitrogen concentrations as a proxy for ammonia consumption in both systems were compared with Student’s *t*-test for conditions 2 and 3 while condition 1 required the systems to be compared with Welch’s *t*-test due to unequal variance between the ammonia- and urea-fed systems. The data distribution and variance for all tests were checked with the Shapiro–Wilks and Levene tests, respectively. We tested if the microbial community in GAC samples clustered significantly by nitrogen source, concentration, and reactor depth using the Bray–Curtis dissimilarities calculated from the ASV abundance table and applying a PERMANOVA test using the adonis function in the R package vegan v2.6-4 (52). Correlations between microbial community composition and concentrations of ammonia, nitrite, and nitrate measured in the biofiltrations were calculated with the Mantels test. The mean relative abundances of nitrifier ASVs and qPCR-based relative abundances of comammox *Nitrospira*, strict AOB, and *Nitrospira*-NOB were compared in both systems across the nitrogen concentrations using ANOVA. Pearson correlation was used to statistically quantify and test the significance of the relationship between the abundance of comammox *Nitrospira* and *Nitrospira*-NOB in both systems.

## RESULTS

### Nitrogen biotransformation in ammonia- and urea-fed systems

**Fig 1:**
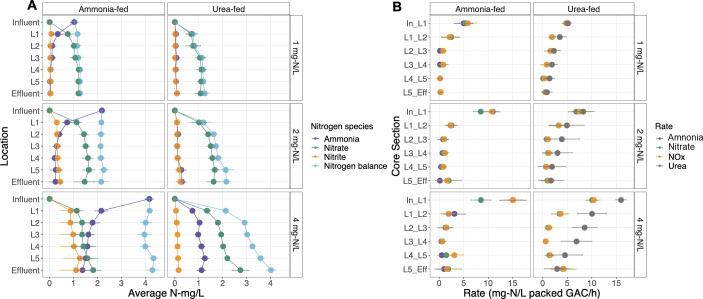
(**A**) Concentrations of ammonia, nitrite, and nitrate measured at five depths along the reactors. Measurements were taken at 1.27 (**L1**), 2.54 (**L2**), 3.81 (**L3**), 5.08 (**L4**), and 6.35 (**L5**) cm from the top of GAC in the reactors. Data points represent the average concentration obtained from the different reactor depths along with error bars for standard deviation. (**B**) Rates of nitrogen biotransformation along the depths of the reactors. Rates are colored by the type of nitrogen biotransformation (purple, ammonia oxidation; green, nitrate production; orange, NO_x_ production; gray, urea degradation). Data points represent the average rates obtained from the different reactor depths along with error bars for standard deviation.

Urea- and ammonia-fed systems had similar concentrations of ammonia, nitrite, and nitrate in the effluent [sample size (*n*) = 16, Wilcoxon *P* > 0.05] (Fig. S2) and similar depth-wise distributions of inorganic nitrogen species (Fig. 1A) at the lowest influent concentration (condition 1: 1 mg-N/L). The majority of the influent nitrogen (~70%) was completely oxidized to nitrate in the topmost portion of the reactors (0–1.27 cm section) (Fig. 1A and B). Rates of ammonia oxidation (4.99 ± 1.95 mg-N/L packed GAC/h) and urea degradation (5.13 ± 0.70 mg-N/L packed GAC/h) were highest in the 0–1.27 cm section and were nearly equal to the rate of nitrate production (5.31 ± 1.55 and 4.79 ± 0.83 mg-N/L packed GAC/h, respectively) (Fig. 1B). Increasing the influent nitrogen concentrations to 2 mg-N/L (i.e., condition 2) led to significantly higher nitrite accumulation in the ammonia-fed reactor compared to the urea-fed reactor (*n* = 16, Wilcoxon *P* < 0.05) due to an imbalance between ammonia oxidation (10.91 ± 1.49 mg-N/L packed GAC/h) and nitrate production (8.51 ± 2.03 mg-N/L packed GAC/h) rates in the In-1.27 cm section of the ammonia-fed system (Fig. 1B). In contrast, the average urea degradation rate (8.25 ± 2.21 mg-N/L packed GAC/h) was nearly equal to the nitrate production rate (6.85 ± 1.67 mg-N/L packed GAC/h) in the top section of the urea-fed reactor. Nitrite accumulation was exacerbated at the higher influent nitrogen concentration (condition 3: 4 mg-N/L) with significantly higher effluent nitrite concentrations in the ammonia-fed system (1.00 mg-N/L) compared to the urea-fed system (0.10 ± 0.04 mg-N/L). The average ammonia oxidation rate (15.02 ± 2.67 mg-N/L packed GAC/h) in the top section of the ammonia-fed system was 1.8 times higher than the nitrate production rate (8.50 ± 2.03 mg-N/L packed GAC/h). The rates of nitrate production in the 0–1.27 cm section were similar between conditions 2 and 3 (8.56 ± 1.47 and 8.50 ± 2.03 mg-N/L packed GAC/h, respectively) in the ammonia-fed system, indicating that maximum rates of nitrate production had been reached. Interestingly, ammonia accumulation in the urea-fed systems resulted in similar effluent ammonia concentrations as observed in the ammonia-fed systems for conditions 1 and 2, suggesting that both reactors had reached their ammonia oxidation capacity across the entire depth of the reactors.

### GAC microbial community composition

**Fig 2:**
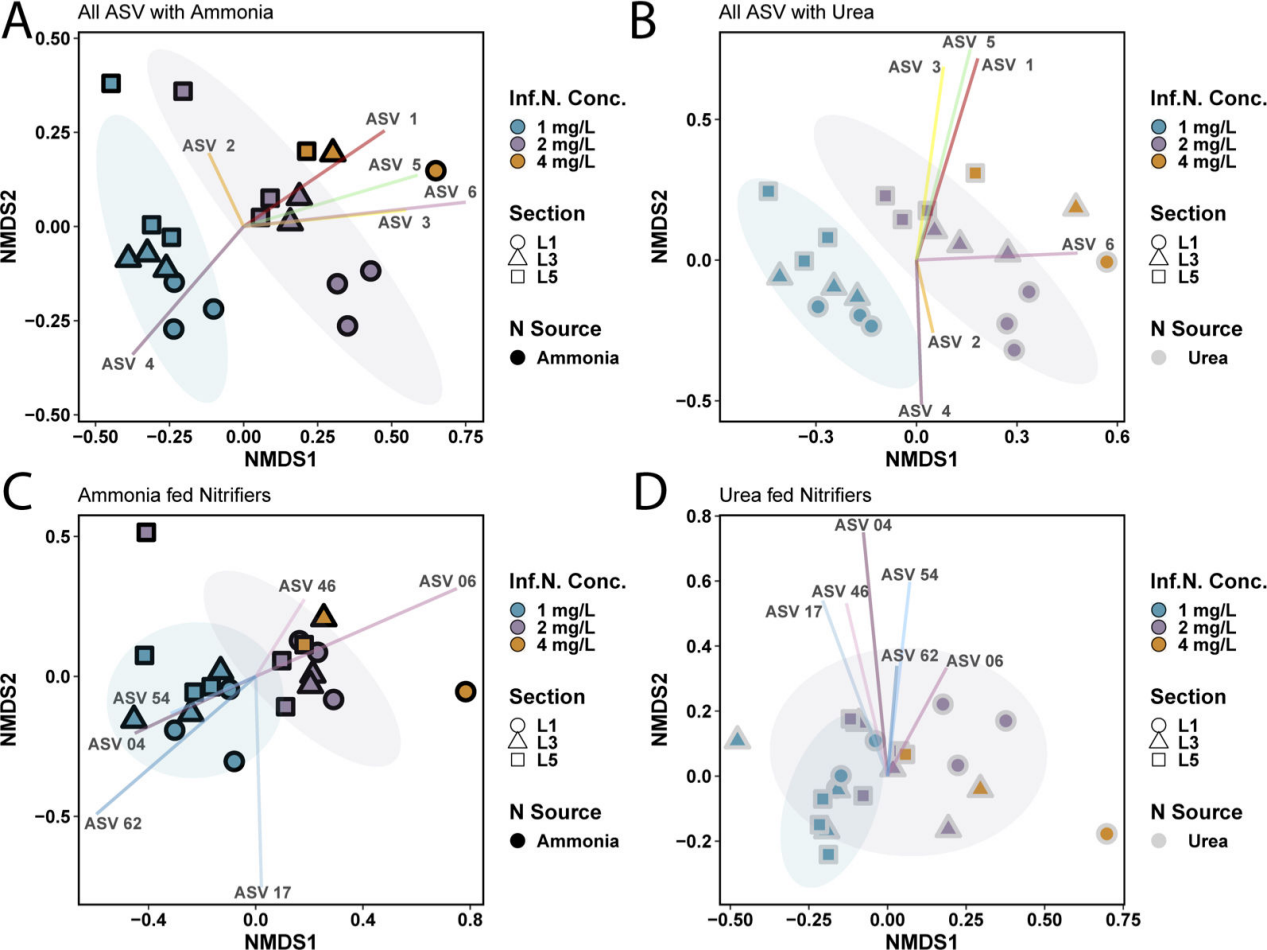
Non-metric multidimensional scaling (NMDS) plots constructed with the abundance tables of all ASVs in the (**A**) ammonia-fed system and (**B**) urea-fed system and nitrifier ASVs in the (**C**) ammonia-fed and (**D**) urea-fed systems. Blue-, purple-, and orange-colored points are GAC samples collected during conditions 1, 2, and 4, respectively. Shape symbolizes the reactor depth the GAC samples were taken from [L1, top (circle); L3, middle (triangle); L5, bottom (square)]. The outline color of shapes represents the system the GAC was collected from (gray, urea-fed; black, ammonia-fed).

Rarefaction to the smallest library size (70,354 reads) resulted in the retention of 1,738 ASVs out of 2,400 constructed from the V4 hypervariable region of the 16S rRNA gene. ASVs with the highest relative abundance belonged to the class *Gammaproteobacteria* (5.09% ± 1.96%: ASV 1 and 4.62% ± 2.96%: ASV 2), *Vicinamibacteria* (3.70% ± 1.49%: ASV 3), *Nitrospira* (3.50% ± 1.63%: ASV 4 and 2.72% ± 1.40%: ASV 6), and *Alphaproteobacteria* (3.40% ± 1.56%: ASV 5). Microbial community composition was shaped significantly by nitrogen concentration (*n* = 42, PERMANOVA *R* = 0.628, *P* < 0.05), nitrogen source (PERMANOVA *R* = 0.134, *P* < 0.05), and reactor section [i.e., GAC sampling point, top (L1), middle (L3), and bottom (L5)] (PERMANOVA *R* = 0.163, *P* < 0.05) (Fig. 2A). Nitrogen source (i.e., ammonia-fed vs urea-fed) played a more significant role in shaping the overall microbial community for condition 2 (*n* = 18, PERMANOVA *R* = 0.224, *P* < 0.05) as compared to condition 1 (*n* = 18, PERMANOVA *R* = 0.104, *P* > 0.05) or condition 3 (*n* = 6, PERMANOVA *R* = 0.412, *P* > 0.05). Community composition of the two reactors may not exhibit a strong difference for condition 1 due to very similar depth-wise nitrogen species profiles (Fig. 1A and B), while differences during condition 3 may not be flagged as significant due to the limited data points. In the ammonia-fed system (Fig. 2A), the microbial community separated into distinct clusters based on nitrogen concentration (*n* = 21, PERMANOVA *R* = 0.614, *P* < 0.05) but not by reactor depth (*n* = 21, PERMANOVA *R* = 0.077, *P* > 0.05). In contrast, both nitrogen concentration (*n* = 21, PERMANOVA *R* = 0.665, *P* < 0.05) and reactor depth (*n* = 21, PERMANOVA *R* = 0.204, *P* < 0.05) were significantly associated with differences in microbial community composition for the urea-fed system (Fig. 2B).

Compositional differences in nitrifier communities were evaluated with ASVs classified as *Nitrospira*-like (nine ASVs) and *Nitrosomonas*-like (five ASVs) bacteria; this is in line with our previous work, which found nitrifiers belonged to only these genera in GAC samples from the same biofiltration system (1), and these were the only nitrifying genera identified in the inoculum used for this study (Table S3). Nitrifier communities in the ammonia- and urea-fed systems were significantly dissimilar during conditions 1 (*n* = 18, ANOSIM *R* = 0.319, *P* < 0.05) and 2 (*n* = 18, ANOSIM *R* = 0.368, *P* < 0.05) (Fig. S2B). Nitrogen source explained a greater variance between communities in condition 3 (*n* = 6, PERMANOVA *R* = 0.406) but was found to be insignificant potentially due to fewer data points. In both the ammonia- and urea-fed systems, nitrifier community composition was most dissimilar between conditions 1 and 3 (*n* = 42, ANOSIM *R* = 0.607 and 0.536, *P* < 0.05) (Fig. 2C and D). Collectively, our results show that the composition of both the whole community and nitrifiers was significantly shaped by nitrogen source and availability. The largest impacts were consistently observed when comparing the lowest and highest nitrogen concentration conditions.

### Impact of nitrogen source and concentrations on nitrifying bacteria

In the ammonia- and urea-fed systems, four *Nitrospira*-like (ASVs 4, 6, 46, and 236) and three *Nitrosomonas-*like (ASVs 17, 54, and 62) ASVs were detected. Mapping ASVs 4, 6, and 46 against *Nitrospira* reference genomes and full-length 16S rRNA sequences indicated that ASV 4 was a *Nitrospira lenta*-like strict NOB (100% ID to NCBI accession number KF724505) and ASVs 6 and 46 were *Nitrospira nitrosa*-like comammox *Nitrospira* (both 100% ID to NZ_CZQA00000000). All *Nitrosomonas*-like ASVs shared high sequence similarity (>98% ID) with 16S rRNA gene sequences within *Nitrosomonas* cluster 6a [*Nitrosomonas ureae* (NZ_FOFX01000070) and Is79 (NC_015731)].

**Fig 3:**
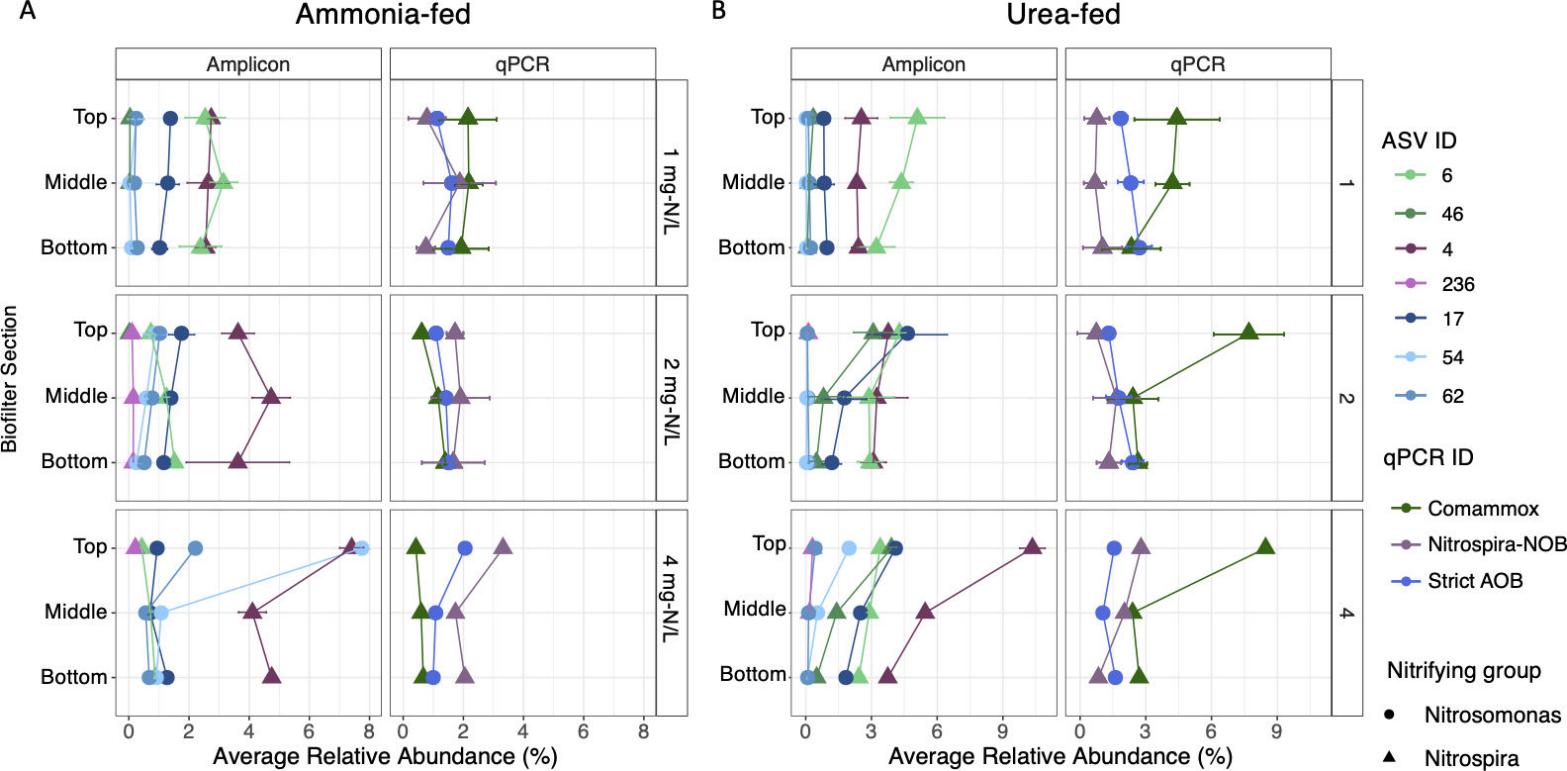
Relative abundance of nitrifier ASVs and qPCR-based relative abundance of comammox *Nitrospira*, strict AOB, and *Nitrospira-*NOB in the (**A**) ammonia-fed and (**B**) urea-fed systems at 1 (top panels), 2 (middle panels), and 4 (bottom panels) mg-N/L. *Nitrosomonas*- and *Nitrospira*-like populations are represented by circles and triangles, respectively. Data points for ASVs are the average relative abundance of nitrifier ASVs calculated in the top, middle, and bottom sections of the reactors during each nitrogen concentration with error bars for standard deviation. Data points for qPCR assays are the average relative abundances of comammox *Nitrospira*, strict AOB, and *Nitrospira*-NOB in the top, middle, and bottom sections of the reactors during each nitrogen concentration with error bars for standard deviation.

In the ammonia-fed system, the relative abundance of *Nitrospira lenta*-like ASV 4 averaged across the reactor (i.e., samples from top, middle, and bottom combined) increased with increasing nitrogen concentrations (1 mg-N/L: 2.63% ±0.45%, 2 mg-N/L: 3.89% ± 1.23%, and 4 mg-N/L: 5.40% ± 1.75%) with its abundance significantly higher in condition 3 compared to condition 1 (*n* = 21, ANOVA/Tukey, *P* < 0.05) (Fig. S3). In contrast, the average relative abundance of comammox*-*like ASV 6 across the column decreased with increased nitrogen concentrations (1 mg-N/L: 2.67% ±0.70%, 2 mg-N/L: 1.16% ± 0.38%, and 4 mg-N/L: 0.67% ± 0.22%) with its the abundance significantly higher during condition 1 compared to both conditions 2 and 4 (*n* = 21, ANOVA/Tukey, *P* < 0.05). Furthermore, the abundance of ASV 6 did not change with depth in the ammonia-fed reactor during all conditions whereas the abundance distribution of ASV 4 appeared to be dependent on nitrogen availability (Fig. 3A). The abundance of ASV 4 was positively associated with concentrations of ammonia (*n* = 21, Pearson *R* = 0.344, *P* < 0.05) and nitrite (*n* = 21, Pearson *R* = 0.304, *P* < 0.05), but the opposite was observed for the abundance of ASV 6, which had a negative association with both ammonia (*n* = 21, Pearson *R* = 0.367, *P* < 0.05) and nitrite (*n* = 21, Pearson *R* = 0.346, *P* < 0.05) (Fig. S4A and B). The relative abundance of *Nitrosomonas-*like ASV 17 across the column remained consistent between conditions (1 mg-N/L: 1.23% ± 0.31%, 2 mg-N/L: 1.44% ± 0.39%, and 4 mg-N/L: 0.97% ± 0.28%) (*P* < 0.05) (Fig. S3) with similar relative abundances found in each section of the reactor regardless of nitrogen concentration (Fig. 3A). ASV 54 replaced ASV 17 as the dominant *Nitrosomonas-*like ASV as its average relative abundance across the column increased 27-fold between conditions 1 (0.12% ± 0.19%) and 4 (3.23% ± 3.88%). The abundance of both ASVs 54 and 62 was positively correlated with the concentration of ammonia (*n* = 21, Pearson *R* = 0.503 and Pearson *R* = 0.474, *P* < 0.05) (Fig. S4A), indicating their abundance increased in response to higher ammonia concentrations in the ammonia-fed system.

While the abundance of *Nitrosomonas-*like ASV 54 increased 40-fold with increased influent nitrogen concentration in the urea-fed system, ASV 17 remained the dominant ASV with its relative abundance increasing from 0.88% ± 0.31% in condition 1 to 2.45% ± 1.92% in condition 3 across the column. In contrast, *Nitrosomonas-*like ASV 62, which increased in abundance proportional to ammonia concentration in the ammonia-fed system, demonstrated no significant change between any of the urea conditions (*n* = 21, ANOVA/Tukey, *P* > 0.05) and remained at low relative abundance, suggesting it was outcompeted in the urea-fed system. While ASVs 4 and 6 were still dominant *Nitrospira*-like ASVs in the urea-fed system, another *Nitrospira*-like ASV (48) increased in abundance across the column from 0.18% ± 0.14% to 1.41% ± 1.36% to 1.93% ± 1.76% for conditions 1, 2, and 3, respectively; ASV 46 was only detected in conditions 1 and 2 in the ammonia-fed system at extremely low abundance (<0.006%), thus showing a clear enrichment in the urea-fed systems (Fig. S3). Its abundance also changed with reactor depth during conditions 2 and 4 in the urea-fed system where its abundance was higher in the top section compared to lower portions (Fig. 3B). Consistent with the ammonia-fed system, the abundance of *Nitrospira lenta*-like ASV 4 was positively associated with ammonia concentrations (*n* = 21, Pearson *R* = 0.289, *P* < 0.05) (Fig. S4C), and that of ASV 6 was negatively associated with nitrite concentrations (*n* = 21, Pearson *R* = 0.398, *P* < 0.05) in the urea-fed system (Fig. S4D).

Comammox *Nitrospira* abundance averaged across the columns based on qPCR assays was significantly lower in conditions 2 (1.06% ± 0.35%) and 3 (0.56% ± 0.13%) compared to its abundance during condition 1 (2.08% ± 0.71%) (ANOVA/Tukey, *P* < 0.05) in the ammonia-fed system (Fig. 3A). Additionally, the relative abundance of comammox *Nitrospira* displayed minimal change along sections of the ammonia-fed reactor during all conditions, which aligns with the trends observed for ASV 6. The relative abundance of *Nitrospira*-NOB averaged across the column assessed by qPCR increased with each nitrogen concentration (1 mg-N/L: 1.14% ± 0.88%, 2 mg-N/L: 1.76% ± 0.73%, and 4 mg-N/L: 2.37% ± 0.84%) though its abundance was not significantly different between each of them (*n* = 42, ANOVA/Tukey, *P* > 0.05). However, its relative abundance was highest overall (3.32%) during condition 3 in the top section where ammonia and nitrite availability was considerably higher compared to the other two nitrogen concentrations. Thus, *Nitrospira*-NOB likely benefited from increased availability of ammonia and nitrite during higher nitrogen concentrations whereas comammox *Nitrospira* preferred the lowest nitrogen condition with limited ammonia availability in the ammonia-fed system. The overall highest abundance of strict AOB and *Nitrospira*-NOB occurred in the top section of the reactor during condition 3.

**Fig 4:**
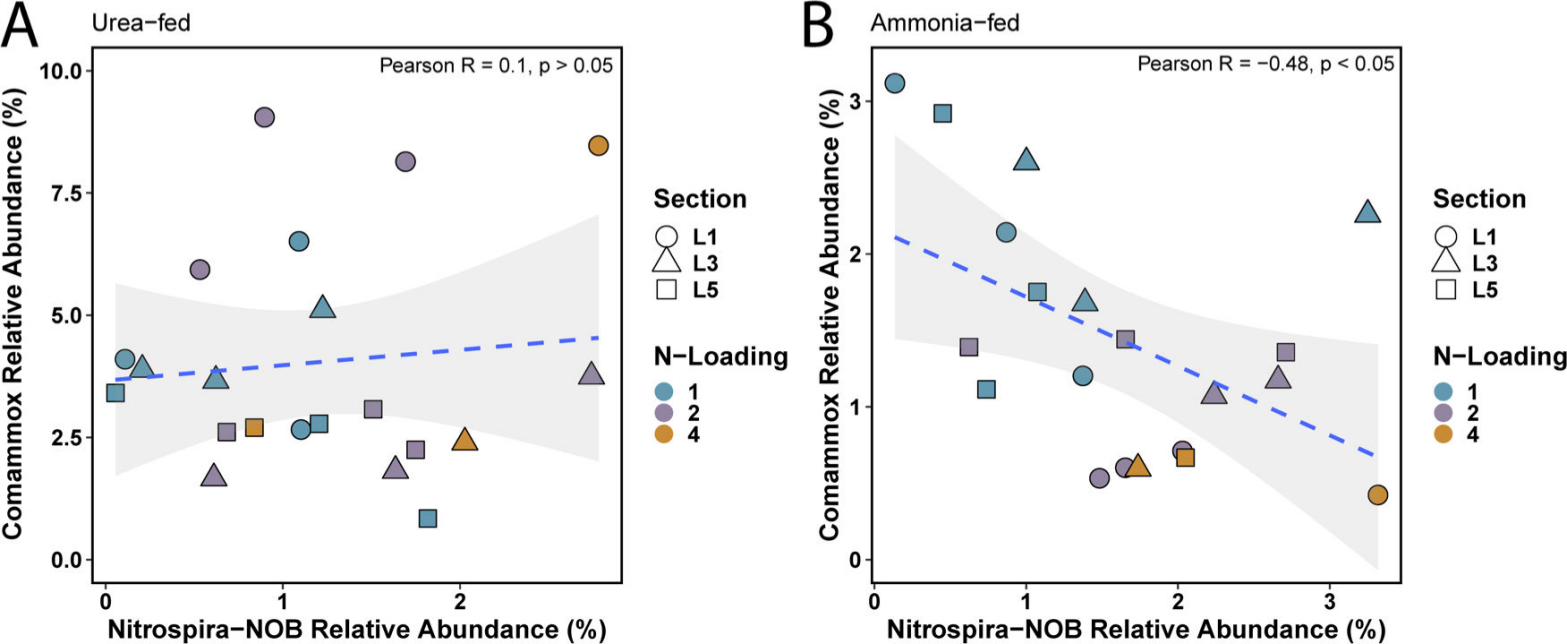
(**A**) Lack of any relationship between the abundance of comammox *Nitrospira* and *Nitrospira*-NOB in the urea-fed system contrasts with (**B**) significant negative association between the abundance of comammox *Nitrospira* and *Nitrospira*-NOB in the ammonia-fed system.

qPCR-based abundance of comammox *Nitrospira* was significantly higher in all urea-fed conditions compared to its abundance in any of the ammonia-fed conditions (Fig. 3A and B), which was also observed for comammox-like ASVs. The combined abundance of comammox-like ASVs was strongly correlated with the qPCR-based abundance of comammox *Nitrospira* (*n* = 42, Pearson *R* = 0.92, *P* < 0.05) (Fig. S5). Furthermore, qPCR- and ASV-based abundances agreed that comammox *Nitrospira* were the dominant ammonia oxidizers regardless of nitrogen concentration in the urea-fed system. The qPCR-based abundance of *Nitrospira*-NOB in the urea-fed system was lower than that of comammox *Nitrospira* during each condition (Fig. 3B). However, the increased abundance of comammox *Nitrospira* did not result in decreased abundance of *Nitrospira*-NOB (*n* = 21, Pearson *R* = 0.10, *P* > 0.05) (Fig. 4A). This is in contrast to the ammonia-fed system where comammox *Nitrospira* and *Nitrospira*-NOB populations demonstrated a significant negative association (*n* = 21, Pearson *R* = −0.48, *P* < 0.05) (Fig. 4B). There are no significant associations between the abundance of comammox *Nitrospira* and strict AOB, and *Nitrospira*-NOB and strict AOB in either the ammonia- or urea-fed systems (data not shown).

### Phylogeny and metabolism of nitrifier MAGs

Two hundred and one hundred seventy-two MAGs were recovered from the metagenomic assemblies from the ammonia- and urea-fed systems, respectively. The nitrifier community in the ammonia-fed system was comprised of three *Nitrosomonas*-like MAGs and two *Nitrospira*-like MAGs (one classified as *Nitrospira*_F and one classified as *Nitrospira*_D), which aligns with the number of dominant nitrifier ASVs in the ammonia-fed system (Table 1). Nitrifier MAGs assembled from the urea-fed system also mirrored the number of dominant nitrifier ASVs with three *Nitrospira*-like (two *Nitrospira*_F and one *Nitrospira*_D) and three *Nitrosomonas*-like MAGs. While an additional *Nitrosomonas*-like MAG was assembled from the urea-fed GAC sample, it was extremely of low quality (completeness <10%).

**TABLE 1:** Quality statistics for nitrifier MAGs assembled from GAC taken from the ammonia- and urea-fed reactors

MAG name	Classification	Reactor	Completeness (%)	Redundancy (%)
Nitrospira_D1_A	Nitrospira_D sp002083555	Ammonia	92.27	3.91
Nitrospira_F1_A	Nitrospira_F	Ammonia	88.88	3.69
Nitrosomonas_1_A	Nitrosomonas	Ammonia	80.3	0.48
Nitrosomonas_2_A	Nitrosomonas sp016708955	Ammonia	97.38	0.51
Nitrosomonas_3_A	Nitrosomonas	Ammonia	92.34	0.03
Nitrospira_F1_U	Nitrospira_F	Urea	92.11	71.93
Nitrospira_D1_U	Nitrospira_D sp002083555	Urea	94.09	4.82
Nitrospira_F2_U	Nitrospira_F sp002083565	Urea	84.65	6.41
Nitrosomonas_1_U	Nitrosomonas	Urea	98.72	0.48
Nitrosomonas_2_U	Nitrosomonas sp016708955	Urea	93.38	0.51
Nitrosomonas_3_U	Nitrosomonas	Urea	93.54	0.03
Nitrosomonas_4_U	Nitrosomonas	Urea	6.22	0

**Fig 5:**
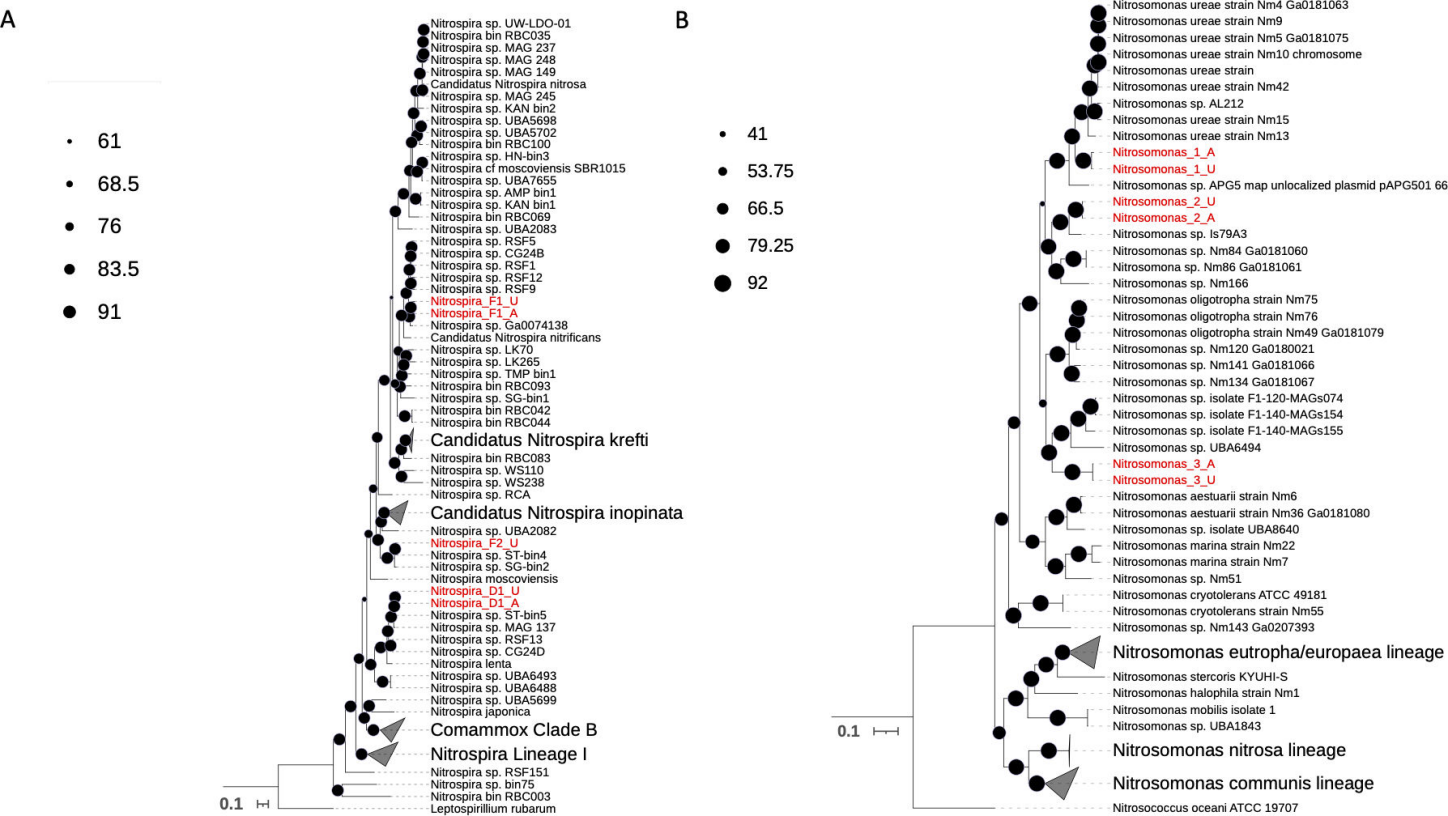
Maximum likelihood trees for (**A**) *Nitrospira* and (**B**) Nitrosomonas based on a set of bacterial single-copy core genes. MAGs assembled in this study are labeled in red, and reference genomes and MAGs are in black.

Phylogenetic analysis with 92 single-copy core genes clustered *Nitrospira*_F1_A with clade A comammox *Nitrospira* (Fig. 5A), and it showed high sequence similarity (~94% ANI) with *Nitrospira* sp. Ga0074138, which is a comammox *Nitrospira* MAG previously assembled by Pinto et al. (9) from GAC obtained from the same reactor. Nitrospira_F1_A shared extremely high sequence similarity (>99% ANI) with Nitrospira_F1_U assembled from the urea-fed system, suggesting that the two MAGs were likely the same population (Fig. S6A). Another *Nitrospira* MAG (Nitrospira_F2_U) assembled from the urea-fed system sample was placed within comammox clade A but clustered separately with other drinking water-related comammox MAGs (*Nitrospira* sp. ST-bin4 and SG-bin2). This MAG shared less than 80% ANI with all other *Nitrospira* MAGs in this study. Phylogenetic placement of hydroxylamine dehydrogenase (hao) gene sequences present in all comammox MAGs in this study was grouped into clade A2 comammox *Nitrospira* (data not shown). The remaining two *Nitrospira* MAGs (Nitrospira_D1_A and Nitrospira_D1_U) clustered with *Nitrospira*-NOB belonging to lineage II (Fig. 5A) with *Nitrospira lenta* and other *Nitrospira*-NOB obtained from a drinking water system (Nitrospira sp. ST-bin5) and rapid sand filter (RSF 13 and CG24D). Nitrospira_D1_A and Nitrospira_D1_U from this study shared over 99% sequence similarity, indicating that they are the same population (Fig. S6A).

ANI comparisons between the *Nitrosomonas*-like MAGs assembled from the ammonia-fed (Nitrosomonas_1_A, Nitrosomonas_2_A, and Nitrosomonas_3_A) and urea-fed (Nitrosomonas_1_U, Nitrosomonas_2_U, and Nitrosomonas_3_U) systems revealed that the same set of three *Nitrosomonas*-like MAGs were assembled from both samples (Fig. S6B). Within-sample ANI comparisons showed that the three *Nitrosomonas*-like MAGs shared less than 95% ANI, suggesting they were separate species. The phylogenomic placement of *Nitrosomonas* MAGs in this study affiliated them with *Nitrosomonas* cluster 6a, which are known for their oligotrophic physiologies (53). Nitrosomonas_1_A and Nitrosomonas_1_U (ANI > 99%) clustered with *Nitrosomonas ureae* while Nitrosomonas_2_A and Nitrosomonas_2_U (ANI > 99%) grouped with *Nitrosomonas* Is79A3 (Fig. 5B). Nitrosomonas_3_A and Nitrosomonas_3_U (ANI > 99%) clustered with uncultured *Nitrosomonas* MAGs that were still within the cluster 6a grouping. Out of all reference comparisons, *Nitrosomonas* MAGs from this study shared the highest similarity to *Nitrosomonas ureae* strain Nm5 Ga0181075 101 (ANI = 83%, Nitrosomonas_1_A and Nitrosomonas_1_U), *Nitrosomonas* Is79 (ANI = 89%, Nitrosomonas_2_A and Nitrosomonas_2_U), and *Nitrosomonas* sp. Nm141 Ga0181066 101 (ANI = 79%, Nitrosomonas_3_A and Nitrosomonas_3_U).

Dereplication of MAGs from urea- and ammonia-fed systems resulted in three Nitrosomonas-like MAGs, one *Nitrospir*a-NOB MAG, and two comammox *Nitrospira*-like MAGs. Filtered reads from the ammonia-fed system were mapped to the set of dereplicated MAGs, revealing that all nitrifier MAGs had 99% breadth of coverage (i.e., percent of genome covered by reads) in both systems. However, the comammox MAG that was assembled only from the urea-fed sample (Nitrospira_F2_U) had very low relative abundance (0.065%) in the ammonia-fed system, which could explain why it was not assembled. Comparably, relative abundances of comammox *Nitrospira* MAGs, Nitrospira_F1_A/Nitrospira_F1_U and Nitrospira_F2_U, were approximately 12- and 5-fold higher in the urea-fed system (~7.81% Nitrospira_F1_A/Nitrospira_F1_U, 0.30% Nitrospira_F2_U) than in the ammonia-fed system (~0.66 Nitrospira_F1_A/ Nitrospira_F1_U, 0.065% Nitrospira_F2_U). These results align with both the qPCR-based abundance of comammox *Nitrospira* and abundance of comammox-like ASVs 6 and 46 in the urea-fed system being substantially higher than their abundance in the ammonia-fed system. Thus, based on abundance trends of the comammox-like ASVs 6 and 46 and comammox MAGs, we associate ASV 6 with the comammox *Nitrospira* population belonging to Nitrospira_F1_A/Nitrospira_F1_U while ASV 46 is associated with Nitrospira_F2_U.

Similar to our previous study, comammox MAGs (Nitrospira_F1_A, Nitrospira_F1_U, and Nitrospira_F2_U) contained genes for urea degradation (*ureCAB*) and transportation (*urtACBCDE*). Strict AOB MAGs Nitrosomonas_1_A and Nitrosomonas_1_U also possessed these genes for ureolytic activity, which aligns with their sequence similarity to and clustering with *Nitrosomonas ureae*. The other *Nitrosomonas* MAGs (Nitrosomonas_2_A, Nitrosomonas_2_U, Nitrosomonas_3_A, and Nitrosomonas_3_U) only encoded a single urea accessory gene (*ureJ*) and gene encoding for urea carboxylase. An unbinned *Nitrosomonas*-associated *ureC* gene was found in the metagenome assembly from the urea-fed system, suggesting that another urease-positive strict AOB MAGs could have been present in the system. *Nitrospira* MAGs (Nitrospira_D1_A and *Nitrospira*_D1_U) did not contain urease genes; however, unbinned genes for *ureA* with 100% sequence ID match to strict NOB *Nitrospira lenta* were detected in the metagenome assembly for both samples, suggesting that *Nitrospira*-NOB were urease-positive.

## DISCUSSION

### Nitrite accumulation in ammonia-fed but not urea-fed system may be associated with NOB inhibition and the rate of ammonia production from urea

Strict AOB and *Nitrospira*-NOB were the dominant nitrifiers in the ammonia-fed systems and particularly at higher ammonia concentrations with the ammonia oxidation rates being consistently higher than the nitrite oxidation rates leading to nitrite accumulation. While nitrite accumulation occurred in the ammonia-fed reactor for condition 3, *Nitrospira*-NOB were more abundant than both AOB and comammox *Nitrospira*. It could be possible that despite their high abundance, *Nitrospira*-NOB were impacted by higher ammonia concentrations of conditions 2 and 3. Fujitani et al. (54) observed that the average *K*_m_ value for nitrite (0.037 mg/L) attributed to a *Nitrospira*-NOB strain originating from a drinking water treatment plant increased fivefold to approximately 0.18 mg-N/L NO_2_^−^ in the presence of free ammonia concentrations around 0.85 mg NH_3_-N/L (54). Thus, decreased nitrite affinity could have impacted the ability of this *Nitrospira* strain to oxidize low nitrite concentrations depending on the concentration of free ammonia. Further, in wastewater systems, suppression of strict NOB activity can be achieved at ammonia concentrations higher than 5 mg-N/L (55). Here, in the ammonia-fed system, average ammonia concentrations observed in the top section of the reactor during conditions 2 (0.89 mg NH_3_/L) and 4 (2.64 mg NH_3_/L) were in line with free ammonia concentrations shown to impact nitrite affinity of *Nitrospira*-NOB strain KM1 in Fujitani et al. (54), thus explaining nitrite accumulation. In the urea-fed system, urease-positive nitrifiers, including comammox *Nitrospira* and *Nitrospira*-NOB, regulated ammonia production and, thus, potentially controlled ammonia availability. While ammonia did accumulate during the highest nitrogen concentration in the urea-fed reactor, unlike the ammonia-fed reactors, comammox *Nitrospira* abundance did not decrease, and there was no nitrite accumulation. This is likely because the highest ammonia concentrations in the urea-fed reactor were consistently lower than the highest concentrations in the ammonia-fed reactors, and thus, comammox *Nitrospira* were not outcompeted by AOB and both comammox and *Nitrospira*-NOB were likely not inhibited.

### Increased ammonia availability in ammonia-fed reactor detrimentally impacted comammox populations

Consistent with the reported higher ammonia affinity (i.e., lower *K*_m(app)_) of comammox *Nitrospira* compared to strict AOB (14–16), comammox *Nitrospira* did indeed dominate over AOB only during the lowest nitrogen concentration in the ammonia-fed system. Though strict AOB were affiliated with *Nitrosomonas* cluster 6a characterized with higher ammonia affinities (*K*_m(app)_ = 0.24–3.6 µM) (53) compared to other AOB, the reported ammonia affinity for comammox *Nitrospira* is still substantially higher for comammox *Nitrospira* (*K*_m(app)_ = 63 nM). In addition to ammonia affinity, it is very likely that ammonia tolerance played a role as the partial inhibition of ammonia oxidation activity by *Ca*. *Nitrospira kreftii* has been reported at ammonia concentrations as low as 0.425 mg/L, which is within the range of ammonia concentrations observed in the ammonia-fed reactor (0.25–2 mg-N/L) during conditions 2 and 3. However, ammonia sensitivity resulting in the partial inhibition of ammonia oxidation has not been observed for *Ca*. *Nitrospira inopinata* (16) and *Ca*. *Nitrospira nitrosa*-like comammox *Nitrospira* in wastewater systems where ammonia concentrations are higher (2, 3, 19). Comammox *Nitrospira* in this study may be adapted to low ammonia concentrations and were most similar to other clade A1 comammox *Nitrospira* obtained from low ammonia environments. Thus, continuous exposure to elevated ammonia concentrations could play a role in the observed reduction in the abundance of comammox *Nitrospira* via inhibition.

### Increase in urea concentration favored *Nitrospira* bacteria including comammox *Nitrospira*

Comammox *Nitrospira* were the dominant nitrifier across all conditions in the urea-fed system with their overall abundance significantly higher in the urea-fed system compared to their abundance in the ammonia-fed system. Favorable conditions under urea-fed conditions for comammox *Nitrospira* were also supported by the highest abundances of comammox *Nitrospira* consistently observed in the top of the urea-fed reactor where urea was most available and the emergence of a second low abundance comammox population in the urea-fed reactor. Our observation is similar to other reports of enrichment of very different comammox populations at much higher urea concentrations (22, 23). Though we are unable to identify the exact reason for comammox *Nitrospira* enrichment on urea, it could be a combination of metabolic traits associated with urea uptake and utilization. Specifically, comammox *Nitrospira* may balance the rate of ammonia production from urea with its ammonia oxidation rate, thus maximizing ammonia availability while also maintaining ammonia concentrations at non-inhibitory levels. Further, additional urea transporters are found in comammox genomes that are absent in other *Nitrospira* including an outer-membrane porin (*fmdC*) for uptake of short-chain amides and urea at low concentrations and a urea carboxylase-related transport (*uctT*) (21). Thus, the enhanced ability to uptake urea and regulate its conversion to ammonia balanced with its ammonia oxidation rates may underpin comammox *Nitrospira* preference for urea. Estimating the kinetic parameters such as comammox *Nitrospira*’s affinity for urea and uptake rate relative to other nitrifiers and ammonia production relative to its own ammonia oxidation rates would be extremely useful for assessing their overall preference for urea.

### Nitrogen source drives potential competitive and co-operative dynamics between aerobic nitrifiers

In these continuous flow reactors and our previous batch microcosm experiments (1), we observed nitrogen source-dependent dynamics between the abundance of comammox *Nitrospira* and *Nitrospira*-NOB. We hypothesized that tight metabolic coupling exists between strict AOB and *Nitrospira*-NOB when urea is supplied due to reciprocal feeding between the two groups. Here, the production of nitrite can be controlled by urease-positive *Nitrospira*-NOB via cross-feeding ammonia to strict AOB, which in turn provide nitrite at a rate at which *Nitrospira*-NOB can consume it. This dynamic between canonical nitrifiers substantially contrasts with their relationship when only ammonia is provided as *Nitrospira*-NOB are fully dependent on strict AOB to provide them nitrite. Therefore, a negative relationship between comammox and canonical NOB *Nitrospira* when only ammonia is available may reflect comammox *Nitrospira* limiting *Nitrospira*-NOB access to nitrite (produced by AOB) by performing complete ammonia oxidation to nitrate. In contrast, at high ammonia concentrations, comammox *Nitrospira* may in fact be a source of nitrite for *Nitrospira*-NOB as their ammonia oxidation rates are faster than their nitrite oxidation rates, and their affinities for nitrite are lower than that of *Nitrospira*-NOB (13, 56). Supplementation with urea eliminates this potential comammox-NOB negative association as both nitrifiers are urease-positive and potentially produce ammonia themselves for different purposes (i.e., comammox produce their own ammonia; strict NOB provide ammonia to strict AOB). Competition for urea would then be determined by the urea affinity and uptake rates, which are currently unknown. However, in this study, we show that the increased abundance of comammox *Nitrospira* did not result in the decreased abundance of *Nitrospira-*NOB in the urea-fed system. This suggests that the apparent competitive dynamics between these nitrifiers is reduced when an alternative nitrogen source is available compared to ammonia which induced a competitive relationship.

In this study, the impact of nitrogen source and availability on nitrifying communities was evaluated in continuous flow column reactors, supplied with either ammonia or urea and operated over three different nitrogen concentrations. Consistent with our previous batch microcosm experiments (1), we show that different nitrogen sources and concentrations distinctly shape the nitrifying community. Direct supply of ammonia favored a combination of AOB and NOB particularly as the nitrogen concentrations were increased, with a decrease in comammox *Nitrospira* abundance likely associated with ammonia-based inhibition. Ammonia availability has been considered an important niche differentiating factor between comammox *Nitrospira* and strict AOB, and here, we show that it may also be a significant factor in shaping populations of comammox *Nitrospira*. In contrast, the urea provision promoted the abundance of multiple comammox populations along with strict AOB and *Nitrospira*-NOB. With urea as a nitrogen source, nitrification can be initiated by urease-positive nitrifiers controlling ammonia production and its availability, which in turn significantly impacted nitrification process performance.

## Data Availability

Raw fastq files for amplicon sequencing and metagenomic sequencing data, metagenomic assembly, and curated MAGs are available via NCBI bioproject submission number PRJNA1027363.

## SUPPLEMENTAL MATERIAL

Supplemental information spectrum.03181-23-s0001.docx Supplemental tables and figures.

ASM does not own the copyrights to Supplemental Material that may be linked to, or accessed through, an article. The authors have granted ASM a non-exclusive, world-wide license to publish the Supplemental Material files. Please contact the corresponding author directly for reuse.
