# Supplementary material for: Nitrogen source influences the interactions of comammox bacteria with aerobic nitrifiers
Source: Microbiol Spectr. 2024 Mar 21;12(5):e03181-23. doi: 10.1128/spectrum.03181-23 (PMC11064514; doi:10.1128/spectrum.03181-23)
Supplement: Supplemental information — Supplemental tables and figures. [file spectrum.03181-23-s0001.docx]

**Supplemental Table 1**: Sequences of primers, PCR reaction and thermocycling conditions, and qPCR efficiencies for quantification of comammox bacteria *amoB*, *Nitrospira* 16S rRNA, AOB 16S rRNA and total bacteria 16S rRNA.

| **Target** | **Primer Set** | **Forward Primer sequence** | **Reverse Primer sequence** | **Final concentration in PCR reaction** | **Annealing Temp (°C) /Time (sec)** | **Amplicon length (bp)** | **qPCR efficiencies** | **Reference** |
| --- | --- | --- | --- | --- | --- | --- | --- | --- |
| Comammox clade A amoB gene | Mod_CMX_amoB 148F/485R | TGGTAYGAYACNSARTGGG | CCNGTGATRTCCATCCA | 0.5 mM F/R | 52/45 | 337 | 90.10 ± 0.81% | (1) |
| Nitrospira 16S rRNA gene | Nspra675F - 746R | GCGGTGAAATGCGTAGAKATCG | TCAGCGTCAGRWAYGTTCCAGAG | 0.5 mM F/R | 58/30 | 93 | 102.50 ± 0.71% | (2) |
| AOB 16S rRNA gene | CTO189FA/B/C-RT1R | GGAGRAAAGCAGGGGATCG+ GGAGGAAAGTAGGGGATCG | CGTCCTCTCAGACCARCTACTG | 0.4 mM F/R | 57/30 | 116 | 96.10 ± 1.13% | (3) |
| Total Bacteria 16S rRNA gene | F515 - R806 | GTGCCAGCMGCCGCGGTAA | GGACTACHVGGGTWTCTAAT | 0.2 mM F/0.4 mM R | 50/15 | 291 | 90.00 ± 0.71% | (4) |

1. Vilardi KJ, Cotto I, Sevillano M, Dai Z, Anderson CL, Pinto A. 2022. Comammox *Nitrospira* bacteria outnumber canonical nitrifiers irrespective of electron donor mode and availability in biofiltration systems. FEMS Microbiology Ecology 98.

2. Graham DW, Knapp CW, Van Vleck ES, Bloor K, Lane TB, Graham CE. 2007. Experimental demonstration of chaotic instability in biological nitrification. ISME J 1:385–393.

3. Hermansson A, Lindgren P-E. 2001. Quantification of Ammonia-Oxidizing Bacteria in Arable Soil by Real-Time PCR. Applied Environmental Microbiology67:972–976.

4. Caporaso JG, Lauber CL, Walters WA, Berg-Lyons D, Lozupone CA, Turnbaugh PJ, Fierer N, Knight R. 2011. Global patterns of 16S rRNA diversity at a depth of millions of sequences per sample. Proceedings of the National Academy of Sciences 108:4516–4522.

**Supplemental Table 2**: Details of gBlock qPCR standards and dilution range for 7-point standard curve (1 order of magnitude difference between points).

| **Target gene** | **Sequence source** | **Gblock sequence** | **Standard curve range (copies/5 µl)** |
| --- | --- | --- | --- |
| Comammox clade A amoB | Nitrospira inopinata | CACCGTGAATTGGTATGACACTGAATGGGTGGGGAAAAGCACTGCGGTAAATGATGTTACATACATGAGGGGCAAGTTTCATCTGTCTGAAGACTGGCCTCGTGCGGTAGTGAAACCCCATCGAACGTTCGTCAATGTCGGCTCTCCTAGCTCCGTCTTTGTGCGGTTAAGCACGAAGGTTGGTGGGGTGCCGATGTTTGTGTCTGGTCCTATGGAAATCGGGCGTGATTATGAATATGAGATCACGTTGAAGGCGAGACTTCCTGGACATCATCACATTCACCCTATGTTTTCTGTTAAAGAGGCTGGTCCCATTGCCGGACCGGGTGGGTGGATGGATATCACGGGCCGATACGCT | 10^2^-10^8^ |
| Nitrospira 16S rRNA/Total bacteria 16S rRNA | Nitrospira inopinata | GGCTAACTTCGTGCCAGCAGCCGCGGTAATACGAAGGTGGCAAGCGTTGTTCGGATTTACTGGGCGTACAGGGAGCGTAGGCGGTTGGGTAAGCCCTCCGTGAAATCTCCGGGCCTAACCCGGAAAGTGCGGAGGGGACTGCTTGGCTAGAGGATGGGAGAGGAGCGCGGAATTCCCGGTGTAGCGGTGAAATGCGTAGAGATCGGGAGGAAGGCCGGTGGCGAAGGCGGCGCTCTGGAACATTTCTGACGCTGAGGCTCGAAAGCGTGGGGAGCAAACAGGATTAGATACCCTGGTAGTCCACGCCCTAAA | 10^2^-10^8^ (Nitrospira) 10^3^-10^9^ (Total Bacteria) |
| AOB 16S rRNA gene | Nitrosomonas europaea | CATATCTCTGAGGAGAAAAGCAGGGGATCGCAAGACCTTGCGCTAAAGGAGCGGCCGATGTCTGATTAGCTAGTTGGTGGGGTAAAGGCTTACCAAGGCAACGATCAGTAGTTGGTCTGAGAGGACGGCCAACCACA | 10^2^-10^8^ |

**Supplemental Table 3**: Relative abundance of nitrifying bacteria in the inoculum.

| **Amplicon sequence variant** | **Nitrifying group** | **Relative abundance (%)** |
| --- | --- | --- |
| 4 | Nitrospira | 0.11 |
| 6 | Nitrospira | 0.07 |
| 46 | Nitrospira | 0.00 |
| 236 | Nitrospira | 0.00 |
| 17 | Nitrosomonas | 0.04 |
| 54 | Nitrosomonas | 0.01 |
| 62 | Nitrosomonas | 0.01 |
| 180 | Nitrosomonas | 0.01 |
| 301 | Nitrosomonas | 0.00 |

**Supplemental Table 4**: Microbial community composition of the ammonia and urea fed systems based on amplicon sequencing data. The fractional relative abundance is shown at the phylum level with all phyla constitution less than 0.01% to the overall reads combined into the “others” category.

**Ammonia -fed biofilters.**

|  | **Proteobacteria** | **Bacteroidota** | **Planctomycetota** | **Acidobacteriota** | **Nitrospirota** | **Chloroflexi** |
| --- | --- | --- | --- | --- | --- | --- |
| **Inoculum** | 4.8E-01 | 1.3E-01 | 9.5E-02 | 9.0E-02 | 7.5E-02 | 3.0E-02 |
| 1 mg-N/L – L1 | 6.2E-01 | 5.5E-02 | 6.0E-02 | 9.4E-02 | 5.3E-02 | 9.9E-03 |
| 1 mg-N/L – L3 | 5.3E-01 | 7.3E-02 | 8.6E-02 | 1.2E-01 | 5.8E-02 | 1.7E-02 |
| 1 mg-N/L – L5 | 5.1E-01 | 8.6E-02 | 7.9E-02 | 9.9E-02 | 4.9E-02 | 5.0E-02 |
| 2 mg-N/L – L1 | 6.0E-01 | 1.1E-01 | 1.0E-01 | 2.5E-02 | 4.1E-02 | 2.3E-02 |
| 2 mg-N/L – L3 | 5.2E-01 | 9.7E-02 | 8.8E-02 | 7.6E-02 | 5.2E-02 | 3.4E-02 |
| 2 mg-N/L – L5 | 5.6E-01 | 1.4E-01 | 8.7E-02 | 2.6E-02 | 5.5E-02 | 2.4E-02 |
| 4 mg-N/L – L1 | 4.6E-01 | 2.1E-01 | 8.1E-02 | 1.9E-02 | 8.0E-02 | 2.9E-02 |
| 4 mg-N/L – L3 | 4.8E-01 | 1.5E-01 | 9.6E-02 | 4.1E-02 | 4.8E-02 | 2.4E-02 |
| 4 mg-N/L – L5 | 5.0E-01 | 1.3E-01 | 9.3E-02 | 5.1E-02 | 5.6E-02 | 2.6E-02 |

|  | **Gemmatimonadota** | **Myxococcota** | **Verrucomicrobiota** | **Actinobacteriota** | **Latescibacterota** | **Others** |
| --- | --- | --- | --- | --- | --- | --- |
| **Inoculum** | 1.8E-02 | 2.7E-02 | 6.7E-03 | 1.5E-02 | 1.2E-02 | 1.8E-02 |
| 1 mg-N/L – L1 | 2.3E-02 | 4.8E-03 | 4.7E-03 | 2.4E-02 | 1.5E-02 | 3.6E-02 |
| 1 mg-N/L – L3 | 2.9E-02 | 1.2E-02 | 7.7E-03 | 1.6E-02 | 2.2E-02 | 2.2E-02 |
| 1 mg-N/L – L5 | 2.5E-02 | 1.3E-02 | 1.1E-02 | 1.3E-02 | 1.9E-02 | 4.6E-02 |
| 2 mg-N/L – L1 | 2.7E-02 | 3.1E-02 | 7.5E-03 | 1.1E-02 | 8.2E-03 | 2.2E-02 |
| 2 mg-N/L – L3 | 3.2E-02 | 2.6E-02 | 1.6E-02 | 8.9E-03 | 1.6E-02 | 3.0E-02 |
| 2 mg-N/L – L5 | 2.2E-02 | 3.3E-02 | 1.1E-02 | 1.7E-02 | 7.3E-03 | 2.0E-02 |
| 4 mg-N/L – L1 | 1.8E-02 | 3.4E-02 | 1.7E-02 | 2.8E-02 | 7.1E-03 | 1.6E-02 |
| 4 mg-N/L – L3 | 3.2E-02 | 3.5E-02 | 3.5E-02 | 2.4E-02 | 1.0E-02 | 1.8E-02 |
| 4 mg-N/L – L5 | 3.4E-02 | 2.7E-02 | 3.0E-02 | 1.7E-02 | 1.2E-02 | 1.7E-02 |

**Urea -fed biofilters.**

|  | Proteobacteria | Bacteroidota | Planctomycetota | Acidobacteriota | Nitrospirota | Chloroflexi |
| --- | --- | --- | --- | --- | --- | --- |
| **Inoculum** | 4.8E-01 | 1.3E-01 | 9.5E-02 | 9.0E-02 | 7.5E-02 | 3.0E-02 |
| 1 mg-N/L – L1 | 5.6E-01 | 6.5E-02 | 7.7E-02 | 1.1E-01 | 7.9E-02 | 1.5E-02 |
| 1 mg-N/L – L3 | 5.2E-01 | 7.2E-02 | 8.7E-02 | 1.3E-01 | 6.8E-02 | 1.7E-02 |
| 1 mg-N/L – L5 | 5.4E-01 | 6.9E-02 | 7.4E-02 | 1.3E-01 | 5.6E-02 | 2.8E-02 |
| 2 mg-N/L – L1 | 5.4E-01 | 9.4E-02 | 9.8E-02 | 3.9E-02 | 1.1E-01 | 2.4E-02 |
| 2 mg-N/L – L3 | 5.5E-01 | 8.2E-02 | 9.6E-02 | 6.0E-02 | 6.9E-02 | 2.2E-02 |
| 2 mg-N/L – L5 | 5.1E-01 | 9.1E-02 | 9.2E-02 | 8.8E-02 | 6.5E-02 | 3.2E-02 |
| 4 mg-N/L – L1 | 4.3E-01 | 1.3E-01 | 1.2E-01 | 3.1E-02 | 1.8E-01 | 3.0E-02 |
| 4 mg-N/L – L3 | 4.5E-01 | 1.5E-01 | 1.2E-01 | 4.3E-02 | 9.9E-02 | 3.0E-02 |
| 4 mg-N/L – L5 | 4.8E-01 | 1.2E-01 | 9.9E-02 | 7.8E-02 | 6.6E-02 | 3.5E-02 |

|  | **Gemmatimonadota** | **Myxococcota** | **Verrucomicrobiota** | **Actinobacteriota** | **Latescibacterota** | **Others** |
| --- | --- | --- | --- | --- | --- | --- |
| **Inoculum** | 1.8E-02 | 2.7E-02 | 6.7E-03 | 1.5E-02 | 1.2E-02 | 1.8E-02 |
| 1 mg-N/L – L1 | 2.2E-02 | 6.8E-03 | 5.8E-03 | 1.9E-02 | 1.7E-02 | 2.0E-02 |
| 1 mg-N/L – L3 | 2.3E-02 | 1.2E-02 | 7.9E-03 | 1.7E-02 | 2.0E-02 | 2.4E-02 |
| 1 mg-N/L – L5 | 2.2E-02 | 1.4E-02 | 1.1E-02 | 1.4E-02 | 1.9E-02 | 2.7E-02 |
| 2 mg-N/L – L1 | 2.3E-02 | 1.6E-02 | 1.1E-02 | 1.1E-02 | 7.3E-03 | 2.3E-02 |
| 2 mg-N/L – L3 | 2.9E-02 | 3.0E-02 | 2.1E-02 | 7.6E-03 | 1.2E-02 | 2.4E-02 |
| 2 mg-N/L – L5 | 2.9E-02 | 2.1E-02 | 1.7E-02 | 9.0E-03 | 1.6E-02 | 2.6E-02 |
| 4 mg-N/L – L1 | 2.4E-02 | 1.3E-02 | 1.4E-02 | 7.1E-03 | 7.9E-03 | 1.3E-02 |
| 4 mg-N/L – L3 | 2.8E-02 | 1.9E-02 | 2.8E-02 | 5.4E-03 | 7.0E-03 | 2.1E-02 |
| 4 mg-N/L – L5 | 2.9E-02 | 1.7E-02 | 2.5E-02 | 6.5E-03 | 1.4E-02 | 2.3E-02 |


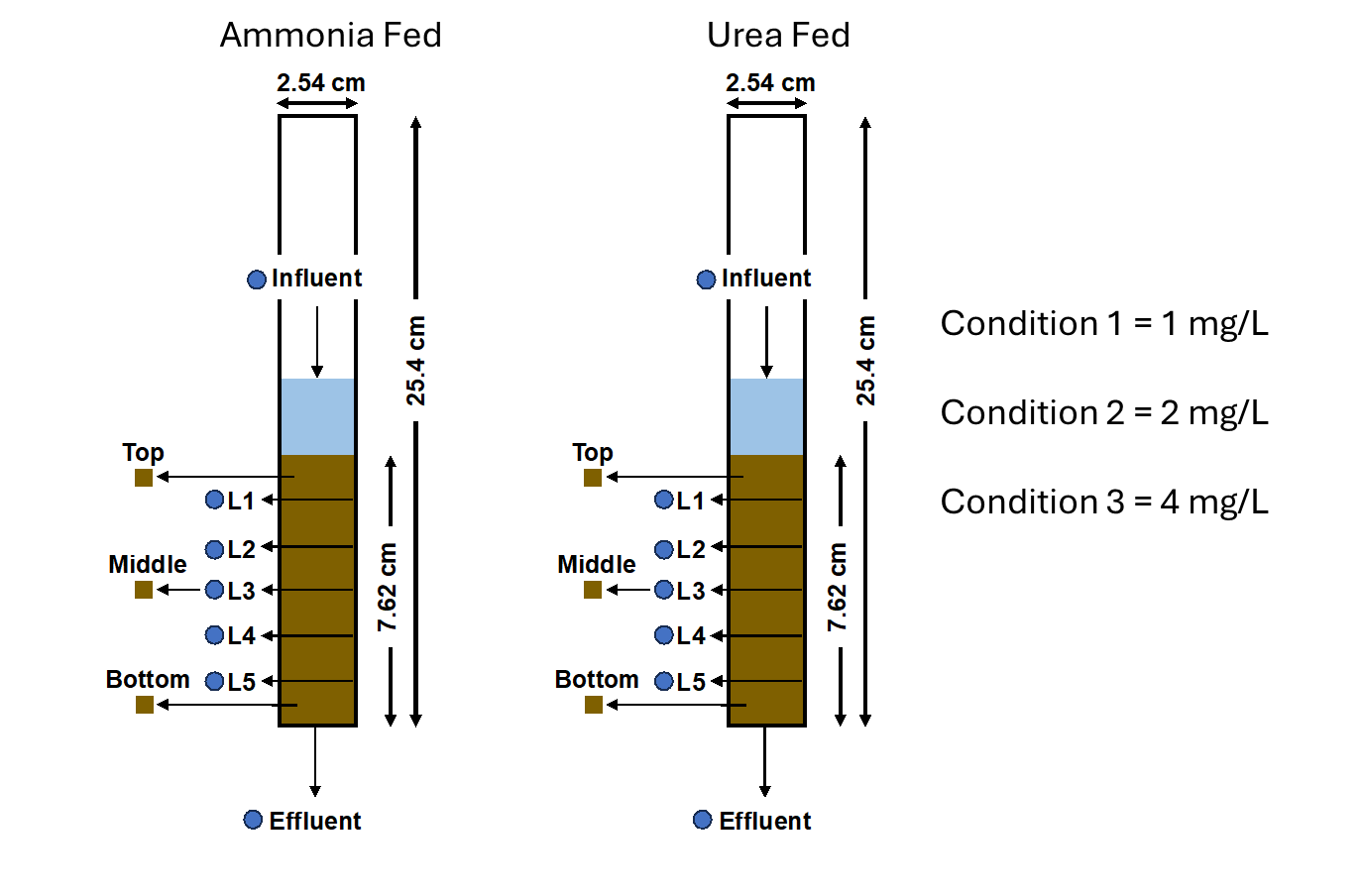


**Supplemental Figure 1**: Schematic of the column reactors used for these experiments. Two identical columns packed with GAC media were operated by supplying either ammonia or urea amended synthetic ground water. Aqueous samples were collected at the influent, effluent, and five sampling ports (L1-L5) that were 1.27 cm from the top and from each other; blue circles indicate locations of aqueous samples. These samples were subject to range of water chemistry analyses, including quantification of inorganic nitrogen species. GAC media samples were collected from the top, middle, and bottom for the column for microbial community analyses; brown squares indicate locations for GAC media sampling.

**Supplementary Figure 2:** Effluent concentrations of ammonia (purple), nitrite (orange), and nitrate (green) measured twice per week in the ammonia- and urea-fed biofiltration systems during all nitrogen loading conditions. Facet labels indicate input inorganic nitrogen concentration.

**Supplementary Figure 3**: Average relative abundance of nitrifier ASVs in the ammonia- and urea-fed biofiltration systems during each condition (black data points) and associated variance (represented by error bar) across the three sampling locations in each column. Each colored data point is an average of technical sequencing replicates. *Nitrospira* and *Nitrosomonas* ASVs are outlined in purple and blue, respectively.

**Supplemental Figure 4**: Significant correlations between the abundance of nitrifier ASVs and concentrations of ammonia (A) and nitrite (B) measured in the ammonia-fed system and concentrations of ammonia (C) and nitrite (D) measured in the urea-fed system.

**Supplemental figure 5**: Significant positive correlation between the abundance of comammox bacteria assessed with qPCR and amplicon sequencing detected using Pearson correlation.

**Supplemental Figure 6:** Average nucleotide identities of (A) *Nitrospira* MAGs and (B) *Nitrosomonas* MAGs recovered in this study.
